# The preparticipation evaluation of the young athlete—an update to what the orthopedic surgeon needs to know

**DOI:** 10.3389/fspor.2025.1650463

**Published:** 2025-10-21

**Authors:** Grace C. Plassche, Daniel James Miller, Robert A. Christian, David P. Trofa, Lauren H. Redler, William N. Levine, Christopher S. Ahmad, Charles A. Popkin

**Affiliations:** ^1^ Department of Orthopedic Surgery, New York-Presbyterian/Columbia University Irving Medical Center, New York, NY, United States; ^2^ Department of Orthopedic Surgery, University of Minnesota Twin Cities, Minneapolis, MN, United States; ^3^ Department of Pediatric Orthopedic Surgery, Gillette Children's, St. Paul, MN, United States

**Keywords:** preparticipation evaluation, RED-S, preparticipation athlete screening, orthopedic surgeon, athlete safety, injury prevention

## Abstract

The preparticipation evaluation (PPE) continues to be a recommended and widely used tool in identifying athletes with health conditions or injury risks that may impact their athletic participation. In the last 10 years, updates to the PPE mirror the increasing impacts of sport specialization, level of competition, importance of mental health in injury risk and recovery, research on cardiovascular and concussive health in youth athletes, and increased awareness of metabolic demands placed on both male and female athletes. The PPE at this snapshot in time exists within an evolving landscape of recommendations. However, it continues to present a vital opportunity for health care providers, preferably within the athlete's “medical home”, to discuss anticipatory guidance, intervene on injury risk, and establish a baseline for future monitoring as the athlete enters competition. As mentioned, there continues to be a need for further research and development, but the orthopedic surgeon should be aware of the purpose the PPE currently serves given the intersectionality of their field with young athletes at the brink or in the midst of injury.

## Introduction

It has been 10 years since we published our recommendations for the orthopedic surgeon in the preparticipation evaluation (PPE) of the young athlete (1). Since then, the number of children and adolescents participating in sports in the United States has nearly doubled to 60 million (2). Along with this tremendous rise comes the trend of early sports specialization, with more youth athletes committing to a single sport prior to the age of 14 (3). Sports-related injuries continue to contribute heavily to the overall pediatric injury burden, with 3.5 million injuries per year resulting in time lost from sport (4). As previously outlined, the PPE is a multisystem evaluation of a youth athlete that has three major components intended to (1) detect health or injury risks that should delay their participation in sport, (2) identify previous injury patterns to prevent recurrence, and (3) provide individualized anticipatory guidance and counseling.

Through collaboration between the American Academy of Family Physicians, American Academy of Pediatrics, American College of Sports Medicine (ACSM), American Medical Society for Sports Medicine, American Orthopaedic Society for Sports Medicine, and American Osteopathic Academy of Sports Medicine the formal PPE is in its 5th iteration as of 2021. There remains no universal mandate regarding the PPE, but it continues to be required by the National Collegiate Association (NCAA) within 6 months of the start of each season and endorsed by the National Federation of State High School Associations (NFHS) (5).

This article serves to update our previous recommendations for orthopedic surgeons, as well as the multidisciplinary teams, who provide medical coverage spanning from the interscholastic to the collegiate level. These teams include primary care physicians, non-operative sports medicine physicians, athletic trainers, physical therapists, and qualified exercise professionals. By including each of these stakeholders in this conversation, a more universal understanding of what it means to take care of the young competitive athlete may be conveyed. This will result in more comprehensive, seamless care for athletes as they progress through each phase of their sport and require the care expertise of each member of the multidisciplinary team. Key updates include addressing the shift in metabolic and nutritional health associated with the relative energy deficiency in sport syndrome, the number of sport hours per week, joint laxity, jump tests, and psychological evaluation. As our athletes evolve, so to must our evaluations to ensure safe athletic participation in the upcoming season.

Our review will largely focus on the evaluation of young, competitive athletes participating in organized sports. The preparticipation evaluation of the general public prior to participating in physical activity will be explored briefly but will not be discussed at length.

## Organization and timing

As previously recommended, the PPE should take place in advance of athletic participation, with the recommendation generally being between 4 and 8 weeks prior. Approximately 3–14 percent of students require additional evaluation and this time frame allows for any additional testing, rehabilitation, or consultation that is required to avoid delaying competition (5). The updated recommendations from the AAP recommend against completing the PPE in a group or team-based setting, but rather within the athlete's “medical home”. Not only does this draw on the trust and continuity of care that exists between an athlete and their primary care practitioner but also allows for routine healthcare topics to be assessed at the same time (2). Conversely, at the collegiate level, the PPE is generally done by a provider local to the university or institution, and it is unrealistic for each athlete to have their individual PCP oversee the PPE. Thus, it is recommended that the same provider complete the entirety of the evaluation for each athlete in an individualized setting in order to provide the most thorough evaluation.

### Medical history

A complete medical history continues to be the most important tool in the PPE as it can detect up to 88% of general medical conditions and 67%–75% of musculoskeletal conditions (2). The most recent version of the PPE monograph, Version 5 published in 2019, provides updated, simpler, and shorter history forms and should be completed in concordance with the athlete's parent or guardian if they are a minor (2). In addition to a thorough and complete review of symptoms, specific areas should be focused on as detailed below. While we will focus on the recommendations within the authors' country of practice, the international standards as they relate to the ACSM recommendations will be explored briefly at the end of this section.

## Cardiovascular issues

Underlying congenital or acquired cardiac malformation continue to account for the vast majority of sudden deaths in athletes younger than 35 years of age (6). There has been much debate about how best to identify patients who may be at increased risk for sudden cardiac death (SCD). Recommendations from the Choosing Wisely Campaign include not ordering annual electrocardiography or any other cardiac screening test for asymptomatic, low risk patients. Screening is recommended to occur during the PPE at a minimum of every three years. There are four main screening questions, in addition to the formal American Heart Association evaluation that has been updated since our last recommendation to include 14 items consisting of 10 historical factors and four physical examination factors (Table 1) (7). The four main screening questions are as follows (8):

1. Have you ever fainted, passed out, or had an unexplained seizure suddenly and without warning, especially during exercise or in response to sudden loud noises, such as doorbells, alarm clocks, and ringing telephones?
2. Have you ever had exercise-related chest pain or shortness of breath?
3. Has anyone in your immediate family (parents, grandparents, siblings) or other more distant relatives (aunts, uncles, cousins) died of heart problems or had an unexpected sudden death before age 50 years? This would include unexpected drownings, unexplained car accidents in which the relative was driving, or sudden infant death syndrome.
4. Are you related to anyone with HCM or hypertrophic obstructive cardiomyopathy, Marfan syndrome, AC, LQTS, short QT syndrome, Brugada syndrome or CPVT, or a condition requiring implantation of a pacemaker or ICD at younger than 50 years?

**Table 1:** The 14-Element American Heart Association Recommendations for Preparticipation Cardiovascular Screening of Competitive Athletes (7).

Medical History	Yes/No?
**Personal History**	
1. Exertional chest pain/discomfort	
2. Unexplained syncope/near-syncope	
3. Excessive exertional and unexplained dyspnea/fatigue, associated with exercise	
4. Prior recognition of a heart murmur	
5. Elevated systemic blood pressure	
6. Prior restriction from participation in sports	
7. Prior testing for the heart, ordered by a physician	
**Family History**	
8. Premature death (sudden and unexpected, or otherwise) before age 50 years due to heart disease, in 1 relative	
9. Disability from heart disease in a close relative\50 years of age	
10. Specific knowledge of certain cardiac conditions in family members: hypertrophic or dilated cardiomyopathy, long- QT syndrome or other ion channelopathies, Marfan syndrome, or clinically important arrhythmias	
**Physical Examination**	
11. Heart murmur	
12. Femoral pulses to exclude aortic coarctation	
13. Physical stigmata of Marfan syndrome	
14. Brachial artery blood pressure (sitting position)	

## Pulmonary issues

There are no significant updates to our previously published recommendations for pulmonary issues. Asthma is a chronic inflammatory disorder of the airways characterized by bronchial hyperresponsiveness leading to intermittent dyspnea, coughing, and wheezing. It is among the most frequent chronic diseases among children and adolescents in the United States (9). Exercise-induced bronchoconstriction describes the transient narrowing of the airways after exercise that is common even in athletes without a diagnosis of asthma. The prevalence of asthma and exercise induced bronchoconstriction among athletes has been estimated to be between 30% and 70% among elite athletes depending on the type of sports performed (10). As such, the team physician should be familiar with the management of this condition. Peak flow measurements may be recorded at the beginning of the season to serve as a baseline for future asthma exacerbations. Short-acting bronchodilators are the mainstay of treatment for intermittent asthma. Patients should have short-acting bronchodilators available for use at home and at school, and ideally a bronchodilator should be kept with a trainer or coach. Make a referral to primary care and consider a pulmonology consult for athletes requiring long-acting bronchodilators or corticosteroids for asthma control.

## Musculoskeletal health

A full musculoskeletal history includes careful review of prior injuries, including mechanism, severity, treatment, and any resulting disability. Prior injury or surgery is a known risk factor for reinjury of a given body part and should direct detailed physical examination of the affected areas. Several studies have demonstrated that athletes who underwent a knee surgery prior to college require more MRIs and have increased rates of knee injury and knee surgery, with a recent study by Falstrom et al. reported as high as 38% of female soccer players who sustained an ACL injury went on to sustain a second ACL injury (11, 12). Additionally, in a cohort comprised of men's football, women's basketball, soccer, and lacrosse players at the collegiate level, it was found that lower extremity musculoskeletal injuries occurred at a higher rate (50%) in previously concussed athletes compared to those athletes that had no history of concussion (20% <0.01) (13). In recognizing prior injury patterns, practitioners can develop athlete-specific rehabilitation and prevention programs (14).

## Medications and supplements

All medications, including over the counter drugs and supplements should be reviewed for possible adverse effects. Additionally, recent changes in the NCAA regulations of banned medications or supplements should be reviewed (Table 2) (15). Most notably, cannabinoids were removed from the banned substances list in 2023. The physician and athlete should be aware of which medications require documentation of medical necessity, such as methylphenidate for attention deficit/hyperactivity disorder. Using the banned substances list provided, the physician has the opportunity to discuss possible elicit use that would disqualify the athlete. Medications that are required for chronic conditions should additionally be documented for exemption, such as diuretics or rate controlling medications for cardiac conditions. These medications can affect fluid status and should be monitored in the setting of athletic exertion.

**Table 2:** Banned drugs and substances according to the National Athletic Association (15).

Substance	Examples
Stimulants	Amphetamine, Methylphenidate, Modafinil
Anabolic Agents	Androstenedione, DHEA, Testosterone
Beta Blockers (banned for rifle only)	Atenolol, Metoprolol, Propranolol
Diuretics and Masking Agents	Bumetanide, Furosemide, Triamterene
Narcotics	Buprenorphine, Hydromorphone, Oxycodone
Peptide Hormones, growth factors, related substances, mimetics	BPC-157, Growth Hormone, EPO, hCG Ex: Synthroid, Insulin, Forteo not banned
Hormone and Metabolic Modulators	Anti-estrogen, aromatase inhibitors, SERMS
Beta-2 Agonists	Albuterol, Salmeterol

With the exception of those documented as medically necessary by provider.

**Figure 1:**
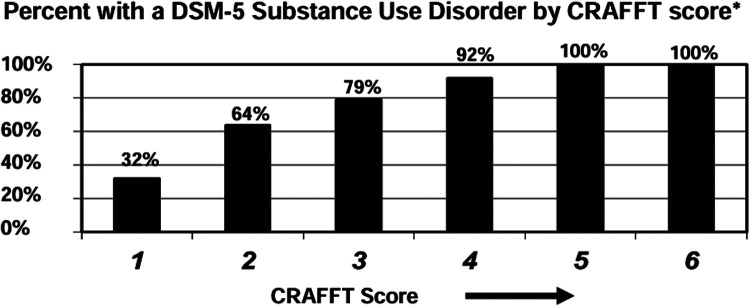
CRAFFT score interpretation: percentage with a DSM-5 substance use disorder by CRAFFT score.

The last decade has seen a decrease in the prevalence of performance-enhancing drug use, though there has been an increase in blood doping amongst youth athletes (16). Reasons for continued use include getting an edge and improve athletic performance by increasing energy, maintaining health and nutrition, and speeding up recovery. Team physicians covering youth sports should be familiar with common ergogenic drugs and supplements, such as anabolic-androgenic steroids (AASs), human growth hormone (hGH), creatine, amphetamines, and erythropoietin (EPO), thus facilitating an open discussion with athletes about the performance benefits of these agents in contrast with the adverse effects and complications that can occur from their use. The PPE in many cases is an appropriate time to ask about ergogenic drug use. The CRAFFT questionnaire (Table 3) (17) is a validated health screening tool that can be used to evaluate for substance use, related driving risk, and possible substance use disorder for youths age 12–21 (18). An affirmative answer to two or more of questions 4–9 suggest a more serious problem and require further assessment by the primary care provider. The graph included here (Figure 1) should be discussed, with motivational interviewing attempted, prior to a referral to psychiatry or addiction medicine if the risk remains high (18). Our previous publication reviews anabolic-androgenic steroids, human growth hormone, creatine, stimulants, and erythropoietin and blood doping in detail and can be used as a guide for understanding the potential usage and adverse effects associated with each.

**Table 3:** CRAFFT Questionnaire (17).

During the Past 12 months on how many days did you:	Number of Days
1. Drink more than a few sips of beer, wine, or any drink containing alcohol? Put “0” if none.	
2. Use any marijuana (weed, oil, or hash, by smoking, vaping, or in food) or “synthetic marijuana” (like “K2,” “Spice”) or “vaping” THC oil? Put “0” if none.	
3. Use anything else to get high (like other illegal drugs, prescription or over-the-counter medications, and things that you sniff, huff, or vape )? Put “0” if none.	
**If you put “0” in ALL of the boxes above, ANSWER QUESTION 4, THEN STOP. If you put “1” or higher in ANY of the boxes above, ANSWER QUESTIONS 4-9.**	**Yes/No?**
4. Have you ever ridden in a CAR driven by someone (including yourself) who was “high” or had been using alcohol or drugs?	
5. Do you ever use alcohol or drugs to RELAX, feel better about yourself, or fit in?	
6. Do you ever use alcohol or drugs while you are by yourself, or ALONE?	
7. Do you ever FORGET things you did while using alcohol or drugs?	
8. Do your FAMILY or FRIENDS ever tell you that you should cut down on your drinking or drug use?	
9. Have you ever gotten into TROUBLE while you were using alcohol or drugs?	

## Allergies

A patient's allergens (both medication and environmental) and the nature of the reaction should still be reviewed. A history of anaphylactic reaction may require the presence of self-administered epinephrine (e.g., EpiPen; Mylan LP) at all activities.

## Metabolic/nutritional health

Screening for the female athlete triad has expanded and evolved over the last decade. The recommendation is now to screen for relative energy deficiency in sport (RED-S), an all-encompassing syndrome first introduced by the International Olympic Committee in 2014, that occurs when energy expenditure is disproportionately high relative to caloric intake (2, 19). Low energy availability is the underlying issue in both the female athlete triad and RED-S and is associated with poor performance, decreased power, delayed recovery and injury resolution in the short term, but in the long term can lead to multi-organ system dysfunction. While the previous screening focused solely on female athletes, RED-S can be evaluated for in all athletes and is characterized by relative energy deficiency, low bone mineral density, impaired psychological, immune, and cardiovascular health. It is reported that RED-S affects 22%–58% of male and female adolescent athletes (20). As with the endocrinopathy seen in the female athlete triad, menstrual dysfunction is an important contributory aspect of RED-S. The equivalent in male athletes is low testosterone levels and erectile dysfunction. Screening for eating disorders, symptoms of low estrogen or low testosterone, and symptoms of low energy such as fatigue or delayed recovery, should be included for both the female and male athlete. A history of stress fractures or overuse injury should raise suspicion for osteopenia or osteoporosis and may warrant laboratory workup or evaluation by an endocrinologist (19). There are conditions that can mimic or mask RED-S, such as pregnancy or iron deficiency anemia, that should be evaluated for as indicated.

## Psychological evaluation

Recently, a cyclical correlation has been described between mental health of the athlete, injury risk, and delayed injury recovery. An AJSM study of NCAA athletes found a significant relationship between anxiety/depressive symptoms during the preseason period and subsequent risk of injury to the athlete, and this has been described in various cohorts from the youth to elite levels (21, 22). The cycle of mental health and injuries extends to post-operative outcomes after sports related injuries with pre-operative mental and physical health scored on the Short-Form Health Survey being predictive of return-to-play after ACL reconstruction and rotator cuff repair (23). This evidence points towards a benefit in being able to identify and potentially intervene with athletes that are at-risk from a mental health perspective. In 2020, the American Medical Society for Sports Medicine released a consensus statement with recommendations for identifying psychological factors as risk factors for poor outcomes after athletic injury (23). Given the correlation between depression/anxiety and risk of injury and/or prolonged recovery, we recommend using two simple screening tools to identify those athletes that are at risk. For depression, the PHQ-9 has been validated in identifying depression (Table 4) (24), while the GAD-7 can be used to screen for anxiety (Table 5) (26). For both scales, a score equal to or greater than 5 is considered mild depression and anxiety respectively, and should trigger the provider to have heightened awareness with these athletes. Additional check-ins and monitoring for worsening of symptoms can be done using the scales provided and a score of 10 or higher on either, indicates the need referral to a mental health professional (27, 28).

**Table 4:** PHQ9 (24).

Over the last two weeks how often have you been bothered by the following problems?	0 Not at all	1 Several Days	2 More than ½ the days	3 Nearly every day
Little interest or pleasure in doing things				
Feeling down, depressed, or hopeless				
Trouble falling or staying sleep, sleeping too much				
Poor appetite or overeating				
Feeling bad about yourself—or that you are a failure or have let yourself or your family down				
Trouble concentrating on things, such as reading the newspaper or watching television				
Moving or speaking so slowly that other people could have noticed—or the opposite, being so fidgety or restless that you have been moving around a lot more than usual				
Mild Depression = 5–10 Moderate Depression = 11–18 Severe Depression = 19–27	Total Score:			
If you checked off any problems above, how difficult have they made it for you to do your work, take care of things at home or get along with other people?	Not difficult at all	Somewhat Difficult	Very Difficult	Extremely Difficult

**Table 5:** GAD7 (25).

Over the last two weeks how often have you been bothered by the following problems?	0 Not at all	1 Several Days	2 More than ½ the days	3 Nearly every day
Feeling nervous, anxious, or on edge				
Not being able to stop or control worrying				
Worrying too much about different things				
Trouble relaxing				
Being so restless that it's hard to sit still				
Becoming easily annoyed or irritable				
Feeling afraid as if something awful might happen				
Total Score:				
If you checked off any problems above, how difficult have they made it for you to do your work, take care of things at home or get along with other people?	Not difficult at all	Somewhat Difficult	Very Difficult	Extremely Difficult

## Sport hours per week

In the last decade, sport specialization has reached a fever pitch among adolescent athletes. Participation in intensive training, focused on a single sport, has become the new norm in the highly competitive youth sports world (29). Both the American Orthopaedic Society for Sports Medicine and the International Olympic Committee have published consensus statements advising against excessive participation in a single sport, specifically in a prepubertal population given concerning physical and mental health concerns including overuse injuries, burnout, and decreased athletic development (29, 30). Despite these prior warnings, the trend of specialization continues, and recent research has confirmed the correlation between overuse injury risk with higher degrees of sport specialization (31). While sport specialization may be inevitable in the older athlete, especially as they approach the collegiate level, there is still concern regarding the sheer number of hours played; A longitudinal case-control study published in the Orthopaedic Journal of Sports Medicine followed athletes aged 7–18 years and found a higher proportion of injuries in athletes that (1) trained more hours per week than their age and (2) had a ratio of training hours to free play hours that exceeded 2:1 (31). Further studies have provided support for the dose response relationship between hours per week and injury risk, specifically 3–7 h per week carried a significantly lower risk than 12 or more hours per week (32). As such, the number of weekly hours spent on their sport, as well as hours of free play per week, is an important screening question to ask youth athletes to establish their future injury risk profile.

## The international perspective

The general medical history that is recommended across international governing bodies in relation to young competitive athletes does not diverge from those previously discussed. For recreational athletes, the 2021 ACSM recommendations reference self-administered Physical Activity Readiness Questionnaire (PAR-Q+), the international standard for risk stratification and screening (33). In response to the call to increase physical activity as a means of managing and preventing chronic disease, the PAR-Q was originally developed in Canada in 1970 and consisted of seven binary questions to evaluate the everyday person prior to engaging in exercise (34). It became widely used as a general screening, but it was only applicable to those ages 15–69 and ultimately had the opposite of the desired effect as it over-screened individuals out of increasing their physical activity (34). Maintaining the initial seven questions, it was revised and expanded with evidence-based consensus to the PAR-Q + in 2010 (Table 6) (34, 35). This instrument has been translated and validated in several difference languages and has allowed a variety of populations to safely start or increase physical activity participation. A recent study out of Brazil highlighted the simple, self-determined clearance pathway that allowed participants to pursue unrestricted activity vs. recommended physician consultation, based on their results (35).

**Table 6:** PAR-Q + screening questions.

Answer the following questions: yes or no	Yes	No
1. Has your doctor said that you have a heart condition or high blood pressure?		
2. Do you feel pain in your chest at rest, during your daily activities of living, or when you do physical activity?		
3. Do you lose balance because of dizziness or have you lost consciousness in the last 12 months? Answer No if your dizziness is associated with over-breathing (including during exercise)		
4. Have you ever been diagnosed with another chronic medical condition (other than heart disease/high blood pressure)? Please list conditions here:		
5. Are you currently taking prescribed medications for a chronic medical condition? Please list conditions and medications here:		
6. Do you currently have (or have had within the past 12 months) a bone, joint, or soft tissue (muscle, ligament, tendon) problem that could be made worse by becoming more physically active? Answer No if you have had a problem in your past, but it does not currently limit your ability to be physically active. Please list conditions here:		
7. Has your doctor ever said that you should only do medically supervised physical activity?		

Both the PPE as discussed here, and the PAR-Q + are tools for assessing physical readiness. However, the PPE is a comprehensive and physician-led clinical exam targeted towards athletes, while the PAR-Q + is a simpler self-screening tool intended to provide general recommendations prior to initiating any physical activity (35). Although the PAR-Q + is an internationally recognized screening tool, it is important to note that an international collaboration of organizations did not recommend any more formal preparticipation evaluation for those intending to be physically active at light to moderate intensity (36). The competitive youth athlete stands to benefit from more rigorous screening than just the PAR-Q + .

### Physical exam

The majority of our recommendations for physical examination of the young athlete from our prior publication remain relevant, with a focus on ensuring that the athlete can safely participate in a sport without the risk of incurring a new or worsening injury. Athletes should still undergo a comprehensive physical examination with the addition of advanced cardiac examination, concussion management and baseline testing, and laboratory evaluation for sickle cell disease as indicated. We provide key updates to our previously published recommendations below and discuss the key difference in international preparticipation recommendations that largely arise within the cardiac evaluation.

## Advanced cardiac evaluation

The cardiovascular physical exam should focus on identifying concerning findings such as pathologic heart murmurs or the clinical findings associated with Marfan syndrome. Our previous recommendations discussed the need for routine advanced cardiac evaluation, exploring the controversies associated with mandated ECG and echocardiography for each athlete (1). While sudden cardiac death (SCD) is relatively rare, with a recent JAMA article citing a nearly 70% decrease in SCD rates between 2002 and 2022 in the NCAA (6), it is still a devastating outcome that must be prevented through thorough screening. As outlined in the medical history, a thorough cardiac history is vital to assessing an athlete's cardiac risk profile, however a recent study published in 2023 by Blank et al. found that, despite 48 states having PPE evaluation forms available online, only 14 included all 14 AHA screening elements (37). The majority of European and international guidelines recommend universal inclusion of ECG while American guidelines, citing high costs and false positive rates, recommend that it be considered only in certain cohorts. Specifically, the 2023 investigation into SCD in NCAA athletes by the American Heart Association found that male athletes carry a 4-fold risk and black athletes a 3-fold risk compared to their counterparts (6). Additionally, a recent four year analysis of all US competitive athletes confirmed trends reported in prior studies that highlighted a significantly higher annual incidence rate of sudden cardiac arrest and sudden cardiac death in male basketball and American football players (38). This highlights the greatest divergence in international recommendations from the American guidelines we focus on in this review and is exemplified by the current recommendations in Italy. A prospective cohort study completed in Veneto (Italy) is the basis for the European Society of Cardiology recommendation that routine, annual screening ECG be done for every athlete (39, 40). The observational study, carried out between 1982 and 2004 reported an 89% decrease in the incidence of SCD in athletes following the implementation of a mandated screening that included an ECG (40). However, the observational nature, lack of multiple control groups, and possible confounding from improved management of athletes across the study period raise concerns about the predictive value of including the ECG routinely (6). While the AHA upholds that ECG should not be included in routine cardiac screening of all athletes, they do point toward possible inclusion for the higher risk athletes previously highlighted and further emphasizes the heightened importance of secondary prevention methods and emergency response protocols within these populations (6). Universally, advanced multimodal imaging such as echocardiography is not recommended. There needs to be close evaluation of the athlete, with referral to cardiology if significant risk is assessed.

## Concussion management and baseline testing

The reported incidence of concussions in young athletes has increased over the last decade, in part due to increased awareness, improved diagnostics, and higher likelihood of symptoms reporting. A study found a 2.2-fold increase in the number of concussions reported in high school athletes in the last decade (41). Recent studies published in JAMA indicate that in children aged 5–7, 43% of concussions are sport-related, increasing to 68% in children aged 8–12 (42). American Football continues to be the leading cause of concussions among young athletes, accounting for 45.3% of those reported from 2012 to 2021 and this does not account for the serious concern for underreporting when it comes to concussions in sport (43). This emphasizes the importance of including concussion management and baseline testing in the PPE.

While concussion can be a difficult diagnosis due to the several biomechanical forces and alteration in mental status, there are several different assessment tools that can be used. Currently baseline concussion testing is recommended by the American Academy of Neurology as well as the US Centers for Disease Control and Prevention in order to establish scores that the athlete can be compared to when there is concern for concussion. A recent JBJS Critical Analysis Review outlines the current status of concussion assessment scales and recommends the King-Devick (KD), child Sport Concussion Assessment Tool (cSCAT3), child Immediate Post-Concussion Assessment and Cognitive Testing cImPACT), and the Vestibular Oculomotor Screening (VOMS) tests to evaluate for concussion in the pediatric athlete. The KD serves as a screening test along with the Standardized Assessment of Concussion (SAC) and the Balance Error Scoring System (BESS) (44).

As the vast majority of concussion symptoms are not easily observable, reported symptoms continue to be the most accurate indicator of concussion as established by a recent JAMA case-control study (45). concussion nondisclosure continues to be a barrier to protecting athletes from the potentially catastrophic sequelae of concussive head impacts. The risks of underreporting symptoms in young athletes range from relapse of concussion symptoms with premature return to sport or return to the classroom to the catastrophic possibility of long-term neuropathologic disorders such as chronic traumatic encephalopathy (CTE) (46, 47). A ground-breaking 2023 JAMA Neurology study found that greater than 40% of contact sport athletes younger than 30 years at the time of death had evidence of CTE, with all participants demonstrating behavioral changes prior to their death (47). It has been reported that one in four athletes experiences pressure to continue to play after a head impact, while nearly half of athletes continue playing with symptoms of a possible concussion (48). Thus, educating youth athletes about possible concussion symptoms, and emphasizing the importance of honest symptom reporting if the athlete does incur a head injury is a vital aspect of this portion of the PPE.

## Jump tests

Historically, the drop box vertical jump test has been widely used to clinically assess ACL injury risk. The athlete would drop from a 1-ft box and maximally jump upon landing. Visual assessment of the knee separation distance, knee flexion, and landing mechanics were used by examiners to classify athletes as high, medium, or low risk of noncontact anterior cruciate ligament injuries (49). However, more recent prospective and 3-D motion analysis studies have suggested that these tests cannot accurately predict injury risk. Specifically, one prospective cohort study of 880 female athletes found that visual assessment of hip and knee control during the drop box vertical jump test, as well as the single-leg squat test, was not associated with accurately predicting anterior cruciate ligament injury risk (50). In most studies, observers over-predicted who might be at risk of an ACL injury. However, there is an argument that given the simplicity of the test and the global benefits that can be derived from the recommended neuromuscular training after a positive test, there still may be utility in completing the drop box vertical jump test (51). A 2022 scoping review of all screening tests for ACL injury highlighted these controversial aspects of jump tests and recommended 3D kinematics and kinetics to increase the utility of the drop box vertical jump test, a notion supported by a 2023 laboratory study published in the Orthopaedic Journal of Sports Medicine (52, 53). However, we recognize that the inclusion of 3D analysis is not feasible for all providers completing the PPE. The drop box vertical jump test continues to demonstrate good to excellent intra-rater reliability and can still be used as a screening test with emphasis on increased knee valgus being associated with future ACL injury (54). Despite its lack of specificity, a positive test can still identify abnormal landing mechanics and help tailor neuromuscular training programs to eliminate asymmetries. However, further research is needed on feasible, multiplanar field-based tests to better evaluate possible deficiencies that can predict injury.

## Laxity

Generalized joint laxity (GJL) is a condition in which synovial joints range beyond normal limits. There has been a lot of discussion regarding the potential risks, and benefits, of GJL in young athletes. GJL allows for greater flexibility, thus potentially benefitting dancers, figure skaters, and gymnasts, with rates of GJH amongst these populations reaching greater than 60% (55). However, several studies have also indicated that GJL increases the risk of musculoskeletal injury and prolongs the recovery time for these athletes (55). Specifically, athletes who participate in contact sports and have GJL are at higher risk for ligamentous injuries (odds ratio 4.7, Pacey et al.) (56, 57). A recent JBJS review highlights the importance of screening patients for GJL using the Beighton and Horan Criteria (Table 7) (55). For young athletes this screening can elucidate a potential risk factor that can be mitigated by joint-stabilizing strength training and injury prevention. We recommend all athletes, both contact and non-contact be screened. A positive Beighton score is ≥6 in pre-pubertal athletes and ≥5 in post-pubertal athletes (58) and should trigger a discussion regarding the risk of injury associated with overtraining joint flexibility rather than stability (59). Physical therapy can be initiated with a focus on joint stability, core strengthening, proper form and biomechanics in proprioceptive and balancing exercises in order to decrease injury risk (60).

**Table 7:** Beighton-Horan criteria for generalized hypermobility (total score/9) (58).

Joint examination		Points
Passive hyperextension of small finger (bilateral)	>90′	1 point for each side
Passive apposition of the thumb to the flexor aspect of the forearm (measured bilaterally)	Thumb touches forearm	1 point for each side
Passive hyperextension of elbow (bilateral)	>10′	1 point for each side
Passive hyperextension of knee (bilateral)	>10′	1 point for each side
Standing trunk flexion w/ knees fully extended	Both palms flat on floor	1 point

### Evidence review of the PPE

Despite the evolving landscape of youth sports and the PPE over the last decade, there have been no major advancements in the evidence base for the PPE. Similar to a decade ago, while the importance of the PPE is universally accepted, the lack of standardization and limited evidence of efficacy persists. While it still stands that only a small percentage of athletes are prevented from participating in sport after the PPE, beyond acting as a screening, it has the potential to act more as an optimization for each athlete and allow for quick action if they face unexpected changes in their physical, mental, and musculoskeletal health. It continues to be widely performed and is mandated by The Special Olympics, most state high school athletic associations and the National Collegiate Athletic Association (2). Corrente et al. recently published a study evaluating the current practice with regards to the MSK screening exam within the PPE and found that while 82% were familiar with the AAP PPE Monograph, only 42% felt that it screened for future injury, while 26% did not perform a physical exam at all (61). This highlights the importance of orthopedic surgeons, and the multidisciplinary teams that collaborate in the care of young athletes, being aware and involved in the PPE. Further emphasized is the need for further research and consistent implementation of evidence-based guidelines. Future establishment of the predictive value for injury prevention of the PPE will require a more standardized, algorithmic approach to its evaluation. Randomized controlled trials comparing the inclusion of various pre-participation screening tools and their implementation in diverse, international settings will aid in establishing the validity of various aspects. Only then will mandates be able to be made regarding its universal use in youth athletics.

### Summary

A decade has passed since our last recommendations on the PPE for orthopedic surgeons and in that time, we have seen an increase in young athletes participating in sport on the scale of millions. This highlights the importance of the PPE now more than ever, especially as young athletes become more specialized, and potentially more prone to orthopedic injury. The orthopedic surgeon should be aware and capable of completing a thorough and complete PPE in order to aid in the care of these young athletes. This review provides key updates to our previous recommendations while highlighting the controversies that continue to exist. The PPE evolves with our population of athletes and still requires further standardization and high-quality validation. However, we can still come together in helping young athletes pursue sport at their desired level by identifying social, behavioral, medical, and musculoskeletal conditions that may prohibit them from doing so.
